# Dengue and Scrub Typhus Coinfection in a Patient Presenting with Febrile Illness

**DOI:** 10.1155/2017/6214083

**Published:** 2017-03-13

**Authors:** Surendra Sapkota, Sudeep Bhandari, Subash Sapkota, Rabin Hamal

**Affiliations:** ^1^Department of Internal Medicine, Tribhuvan University Teaching Hospital, Institute of Medicine, Kathmandu, Nepal; ^2^Tribhuvan University Teaching Hospital, Institute of Medicine, Kathmandu, Nepal

## Abstract

Dengue fever and scrub typhus are common causes of acute febrile illness of unclear origin in Asia. Though coinfections of many vector-borne diseases have been described, articles on dengue and scrub typhus coinfection are distinctly limited. In case of coinfection with dengue and scrub typhus, vigilant monitoring of vitals, platelets transfusion, and timely treatment with doxycycline are necessary. High degree of suspicion has to be made for coinfection in a patient presenting with febrile illness with thrombocytopenia and deranged laboratory parameters in postmonsoon season in endemic regions in Asia.

## 1. Introduction

Dengue fever transmitted by the bite of* Aedes aegypti* mosquito is common in Asian population and often presents as an acute febrile illness of unclear origin.* Orientia tsutsugamushi*, the etiologic agent of scrub typhus, is also a common cause of acute febrile illness of unclear origin in Asia [1]. During the seasonal increase in dengue cases, concomitant scrub typhus infection in endemic areas can cause a diagnostic dilemma. Both diseases have several clinical and laboratory features in common, including rash, thrombocytopenia, and hepatic dysfunction. However, concurrent infection with both pathogens is exceedingly rare, primarily due to the different vectors involved [2]. Here we present a case report of 50-year-old female with acute febrile illness from southern belt of Nepal.

## 2. Case Report

**Figure 1:**
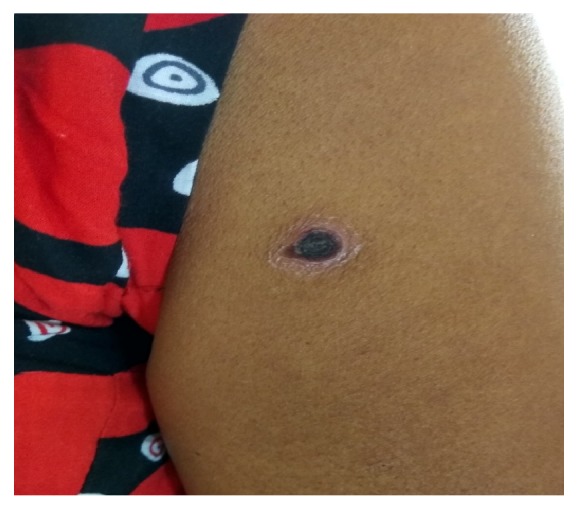
Eschar in right deltoid region.

**Figure 2:**
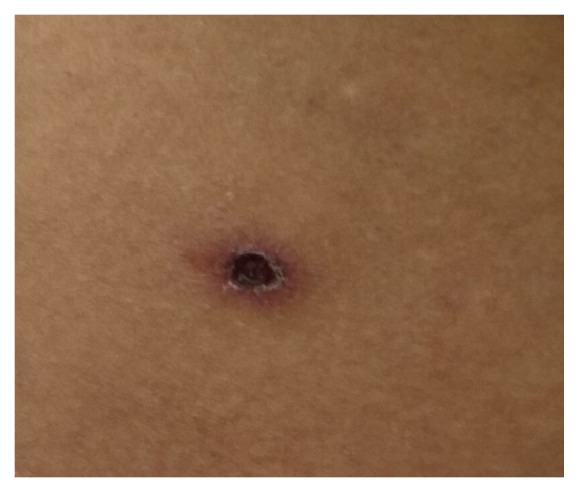
Eschar in right side of the waist.

A 50-year-old married female from Chitwan District presented with fever for 9 days which was high grade, intermittent, measuring up to 104 degrees Fahrenheit and associated with chills, rigors, and sweating. Also she gave history of body ache, weakness, nausea, vomiting, headache, and yellowish discoloration of eyes. She noticed small rash over her right shoulder a few days after first episode of fever which gradually took the shape of cigarette burn eschar. She gave history of regular visit to jungle for cutting wood and grazing cattle. With these symptoms she first visited OPD on 6th day of illness where few tests were run; follow-up was done on 7th day where diagnosis of dengue was made and admitted for one day at local hospital where she received injection ceftriaxone and tablet azithromycin. The investigations revealed low platelets count for which two-pint PRP were transfused. With persistent symptoms and low platelets count, she was referred to our centre the next day. At presentation, she was well oriented to time, place, and person. Her blood pressure was 100/60 mm of Hg, pulse rate was 98 beats per minute, temperature was 100°F, and respiratory rate was 22 per minute. On general physical examination she was icteric with palpable axillary lymph nodes bilaterally and pitting edema was present. Eschar was visible on right deltoid with necrotic centre forming scab and erythematous periphery (Figure 1). Also similar eschar was found on Rt waist (Figure 2). There was hepatosplenomegaly with liver span of 16 cm and spleen was palpable 2 cm below the left costal margin. Occasional crackles were heard in right infra-axillary and inframammary region. Apart from these the other systemic examination revealed no abnormalities. Laboratory investigations revealed leukocytosis and thrombocytopenia. Her LFTs were deranged with raised total bilirubin, direct bilirubin, SGOT, SGPT, ALP, and LDH. Her PT was prolonged. Sodium and potassium were below normal. Values of all these parameters are listed in Table 1. The peripheral blood smear did not show malarial parasite. Serology was nonreactive for HIV, HBS Ag, and HCV. USG showed hepatomegaly with fatty liver grade II and right pleural effusion. CXR showed right pleural effusion. ECG was normal. With these findings empirical treatment was started with iv ceftriaxone and tablet doxycycline. Further reports showed that IgM/IgG antibody for* Leptospira* was negative, IgM/IgG antibody for dengue was positive (method: immunochromatography), and Widal agglutination test for* Salmonella typhi* and* S. paratyphi* was negative. Intravenous fluid and electrolytes were supplemented while oral doxycycline was continued. Report for scrub typhus was awaited. The test result was obtained on 5th day of admission from National Public Health Laboratory, Teku. Scrub typhus was positive (method: ELISA). She was discharged on 7th day of admission and advised for follow-up in a week. But the patient was followed up only after two weeks at local hospital in her hometown where lab investigations were carried out and were normal. She has been doing well since then and this was confirmed by telephone conversation.

## 3. Discussion

The southern Terai region of Nepal is a suitable environment for scrub typhus [4]. Dengue has been found throughout the country from west to east lying in the plain region [5]. Epidemiology and Disease Control Division, Kathmandu, has shared that there were 246 people infected with dengue while 385 with scrub typhus during the period of mid-April to mid-October 2016 [6]. No study has been published on dengue scrub coinfection from Nepal to the best of our knowledge. Scrub typhus and dengue infection were the two most frequently listed presumptive diagnoses in patients who present with fever of unclear etiology at Chiangrai Hospital, Thailand [1]. A study on the etiology of febrile illness among adults in Patan Hospital showed that 4.1% patients (*N* = 876) and 3.2% patients were serologically positive to leptospirosis and scrub typhus, respectively [7] Suspicion of scrub typhus permits early antibiotic treatment, which reduces the mortality of infection with* O. tsutsugamushi* [8]. While dengue test was positive, we suspected coinfection with scrub typhus considering the endemic district she was from, the pathognomonic eschar, and the seasonal factor. Tests for leptospirosis was run as she was at high risk being a farmer who works at rice fields and was also involved with cattle. It is prudent to check for HIV, HBsAg, and HCV in an adult presenting with febrile illness and it is done routinely for admitted cases at our centre. Marked thrombocytopenia, significantly high AST and ALT, and low albumin have been described in coinfections [2]. Oral doxycycline was given at the time of admission with high degree of suspicion for scrub typhus and was still continued after dengue tests came positive. The scrub typhus results came on 5th day of admission. We suggested total of 10 days' course of doxycycline. She was discharged on 7th day of admission. She was followed up through telephone conversation. Treatments of these coinfections pose a great challenge to the treating physicians because of the difficulty in early diagnosis due to overlapping clinical features. Coinfections if not thought of in early days of admission can lead to long duration of hospital stay. Test results take several days to come even in endemic regions affecting our treatment decisions. High degree of clinical suspicion, meticulous physical examination, and knowledge of endemicity are crucial in diagnosing coinfections.

## 4. Conclusion

High degree of suspicion for coinfections has to be made in patients presenting with febrile illness in tropics in postmonsoon season with multisystem involvement and deranged laboratory parameters and not responding to therapy.

## Figures and Tables

**Table 1:** Lab values at the time of admission along with the reference range.

Hematologic	Values	Reference range [3]
TLC	15.4 × 10^9^/L	3.54–9.06 × 10^9^/L
Platelets	35 × 10^9^/L	165–415 × 10^9^/L
Hemoglobin	126 g/L	120–158 g/L
PT	13 sec	12.7–15.4 sec
*Chemistry*		
Glucose (postprandial)	9.6 mmol/L	3.9–6.7 mmol/L
Sodium	128 mmol/L	137–145 mmol/L
Potassium	2.7 mmol/L	2.7–3.9 mmol/L
LDH	1247 U/L	50–150 U/L
Amylase	46 U/L	20–96 U/L
Total protein	57 g/L	60–80 g/L
Albumin	27.3 g/L	40–60 g/L
*Renal*		
BUN	5.5 mmol/L	2.5–7.1 mmol/L
Creatinine	76 *µ*mol/L	44–80 *µ*mol/L
*Hepatic*		
Total bilirubin	129 *µ*mol/L	5.1–22 *µ*mol/L
Direct bilirubin	96 *µ*mol/L	1.7–6.8 *µ*mol/L
ALP	464 U/L	20–90 U/L
SGPT	145 U/L	7–41 U/L
SGOT	130 U/L	12–38 U/L

## References

[1] Watt G., Jongsakul K., Chouriyagune C., Paris R. (2003). Differentiating dengue virus infection from scrub typhus in Thai adults with fever. *American Journal of Tropical Medicine and Hygiene*.

[2] Basheer A., Iqbal N., Mookkappan S. (2016). Clinical and laboratory characteristics of dengue-orientia tsutsugamushi co-infection from a tertiary care center in south India. *Mediterranean Journal of Hematology and Infectious Diseases*.

[3] Longo D. L., Fauci A. S., Kasper D. L., Hauser S. L., Jameson J., Loscalzo J. (2012). *Harrison's Principles of Internal Medicine*.

[4] Brown G. W., Shirai A., Gan E., Bernthal P. (1981). Antibodies to typhus in Eastern Nepal. *Transactions of the Royal Society of Tropical Medicine and Hygiene*.

[5] Annual report of Nepal. http://dohs.gov.np/wp-content/uploads/2014/04/Annual_Report_2070_71.pdf.

[6] The Kathmandu Post Scrub Typhus claims nine in six months. http://kathmandupost.ekantipur.com/news/2016-10-19/scrub-typhus-claims-nine-in-six-months.html.

[7] Murdoch D. R., Woods C. W., Zimmerman M. D. (2004). The etiology of febrile illness in adults presenting to Patan Hospital in Kathmandu, Nepal. *American Journal of Tropical Medicine and Hygiene*.

[8] Silpapojakul K. (1997). Scrub typhus in the western pacific region. *Annals of the Academy of Medicine Singapore*.

